# Targeting lncRNAs in programmed cell death as a therapeutic strategy for non-small cell lung cancer

**DOI:** 10.1038/s41420-022-00982-x

**Published:** 2022-04-04

**Authors:** Yanqin Luo, Jingyang Li, Peng Yu, Jiayi Sun, Yingfan Hu, Xianli Meng, Li Xiang

**Affiliations:** 1 State Key Laboratory of Southwestern Chinese Medicine Resources, College of Pharmacy, Innovative Institute of Chinese Medicine and Pharmacy, Chengdu University of Traditional Chinese Medicine, Chengdu, 611137 P. R. China; 2 School of Preclinical Medicine, Chengdu University, Chengdu, 610106 P. R. China

**Keywords:** Non-small-cell lung cancer, Oncogenesis

## Abstract

Lung cancer is a leading cause of cancer-related mortality worldwide, with non-small cell lung cancer (NSCLC) being the most common histological type. Owing to the limited therapeutic efficacy and side effects of currently available therapies for NSCLC, it is necessary to identify novel therapeutic targets for NSCLC. Long non-coding RNAs (lncRNAs) are non-protein-coding RNAs with a transcript length of more than 200 nucleotides, which play a vital role in the tumorigenesis and progression of multiple cancers, including NSCLC. Induction of programmed cell death (PCD) is the main mechanism leading to tumour cell death in most cancer treatments. Recent studies have demonstrated that lncRNAs are closely correlated with PCD including apoptosis, pyroptosis, autophagy and ferroptosis, which can regulate PCD and relevant death pathways to affect NSCLC progression and the efficacy of clinical therapy. Therefore, in this review, we focused on the function of lncRNAs in PCD of NSCLC and summarized the therapeutic role of targeting lncRNAs in PCD for NSCLC treatment, aiming to provide new sights into the underlying pathogenic mechanisms and propose a potential new strategy for NSCLC therapy so as to improve therapeutic outcomes with the ultimate goal to benefit the patients.

## Facts

- Lung cancer remains the leading cause of cancer-related mortality worldwide, with NSCLC being the most common histological type.
- LncRNAs are non-protein-coding RNAs with a transcript length of more than 200 nucleotides, which play a vital role in the tumorigenesis and progression of NSCLC.
- Diverse forms of PCD pathways participate in the initiation and development of NSCLC, and aberrant regulation of PCD can eventually result in cell carcinogenesis and tumour formation.
- Induction of PCD is the main mechanism leading to tumour cell death in most cancer treatments.
- LncRNAs are closely correlated with PCD including apoptosis, pyroptosis, autophagy and ferroptosis, which can regulate PCD and relevant death pathways to affect NSCLC progression and clinical therapeutic efficacy.
- Targeting lncRNAs is expected to be a promising strategy for lung cancer treatment.

## Open questions

- How does programmed cell death affect the occurrence and development of NSCLC?
- What is the regulatory mechanism of lncRNAs on different types of programmed cell death in NSCLC?
- How to regulate programmed cell death by targeting lncRNA, thus inhibiting the onset and progression of NSCLC?
- How to use the relationship between lncRNA and programmed cell death to promote the sensitivity of NSCLC cells to chemotherapy and radiation therapy, thereby improving patient outcomes?
- How to seek and develop novel drugs targeting lncRNA, especially herbal medicine?

## Introduction

Lung cancer, the second most common type of malignant tumours following breast cancer, remains the leading cause of cancer-related deaths worldwide. According to the data of the International Agency for Research on Cancer (IARC) of the World Health Organization, almost 2.2 million new cases of lung cancer were reported worldwide in 2020, with the mortality ranking the top in all types of cancers [1]. Identified as the major histological category of lung cancer, non-small cell lung cancer (NSCLC) constitutes approximately 85% of lung cancer cases, comprising three main subtypes: adenocarcinoma, squamous cell carcinoma and large cell carcinoma [2]. Because no evident clinical manifestations are observed at the early stage and diagnostic approaches are not effective, more than 75% of patients with NSCLC are diagnosed in the advanced stage, with the 5-year overall survival rate of less than 20% [3]. Despite great advances in the understanding of tumour onset and progression, the underlying mechanisms of NSCLC require further investigation to be comprehensively elucidated. Although currently used treatment approaches for NSCLC have evolved with the introduction of several novel strategies such as molecular target therapy and immunotherapy based on conventional chemotherapy and radiotherapy, the prognosis of many patients is still unfavourable owing to the limited clinical therapeutic efficacy and some unpleasant side effects [4]. Therefore, systematic and in-depth study on the molecular mechanisms of the pathogenesis of NSCLC, identification of new effective therapeutic targets and novel efficacious agents are of great significance for improving patient outcomes and enhancing the quality of life.

Long non-coding RNAs (lncRNAs) are defined as a class of non-protein-coding RNAs with a transcript length of more than 200 nucleotides [5]. They are involved in controlling and regulating various cellular biological processes such as cell proliferation, growth, differentiation, migration and apoptosis via modulation of gene expression and signalling pathways at epigenetic, transcriptional, RNA splicing and post-transcriptional levels by interacting with chromatin, proteins and RNA targets [6, 7]. Recent evidence suggests that abnormally expressed lncRNAs exert a vital role in the tumorigenesis and progression of multiple cancers, including NSCLC [8]. Furthermore, oncogenic and tumour-suppressive lncRNAs have negative or positive impacts, respectively, on tumour formation, proliferation, differentiation, invasion, epithelial–mesenchymal transition, metastasis, cancer stem cell maintenance and drug and radiotherapy resistance in NSCLC [6, 9]. Induction of programmed cell death (PCD) is the primary mechanism of most cancer treatments, which lead to tumour cell death. Studies on molecular mechanisms have demonstrated that lncRNAs are closely correlated with PCD, which include apoptosis, autophagy, ferroptosis and pyroptosis. Furthermore, lncRNAs can regulate PCD and protein expression of relevant death pathways to affect cancer progression and clinical therapeutic efficacy [10]. Therefore, in this review, we summarized the relationship between lncRNAs and four types of PCD (apoptosis, autophagy, pyroptosis and ferroptosis) in NSCLC and clarified the therapeutic role of lncRNAs in PCD for lung cancer treatment, aiming to shed new light on the pathogenic mechanisms of lung cancer and provide a novel therapeutic target for the treatment of lung carcinomas.

## Relationship between lncRNAs and PCD in NSCLC

**Fig. 1:**
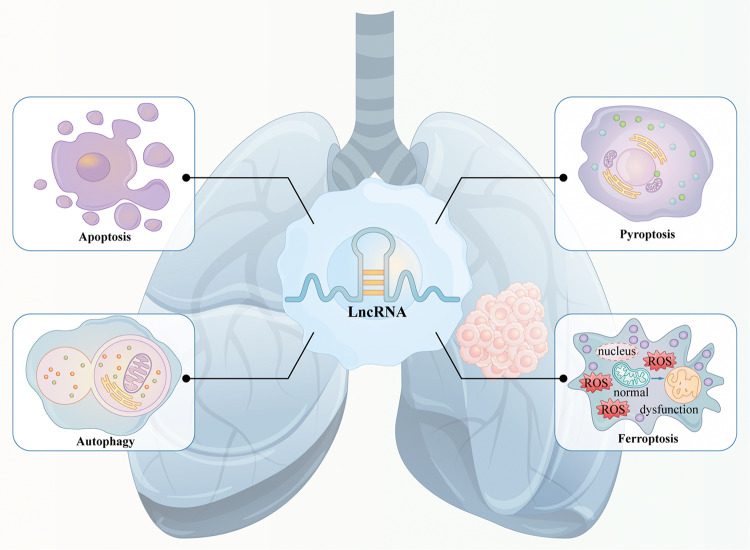
Relationship between lncRNAs and PCD in NSCLC. PCD plays a critical role in the pathogenesis of NSCLC. LncRNAs are strongly associated with PCD, which can regulate different types of PCD and related pathways to influence the tumorigenesis and cancer progression of NSCLC.

PCD refers to a genetically controlled active process that functions during cellular development and in response to stress. It is involved in self-clearance and renewal to maintain homoeostasis and normal physiological activities [11]. Several studies have indicated that PCD plays a critical role in the pathogenesis of NSCLC. Diverse forms of PCD pathways participate in the initiation and development of NSCLC, and aberrant regulation of PCD can eventually result in cell carcinogenesis and tumour formation (Fig. 1). Moreover, PCD pathways are reported to be strongly correlated with resistance in clinical cancer treatment.

Growing evidence has demonstrated that lncRNAs are strongly associated with PCD. LncRNAs regulate different types of PCD by directly influencing the expression of downstream protein molecules and multiple signalling pathways or acting as competing endogenous RNAs (ceRNAs) for binding and sponging target miRNAs to counteract their functions in the tumorigenesis and progression of NSCLC (Fig. 1). The relationship between lncRNAs and PCD provides novel insights into the pathogenic mechanisms of lung carcinomas, and targeting lncRNAs can be exploited as a promising therapeutic strategy for NSCLC treatment.

## LncRNAs regulate apoptosis in NSCLC

**Fig. 2:**
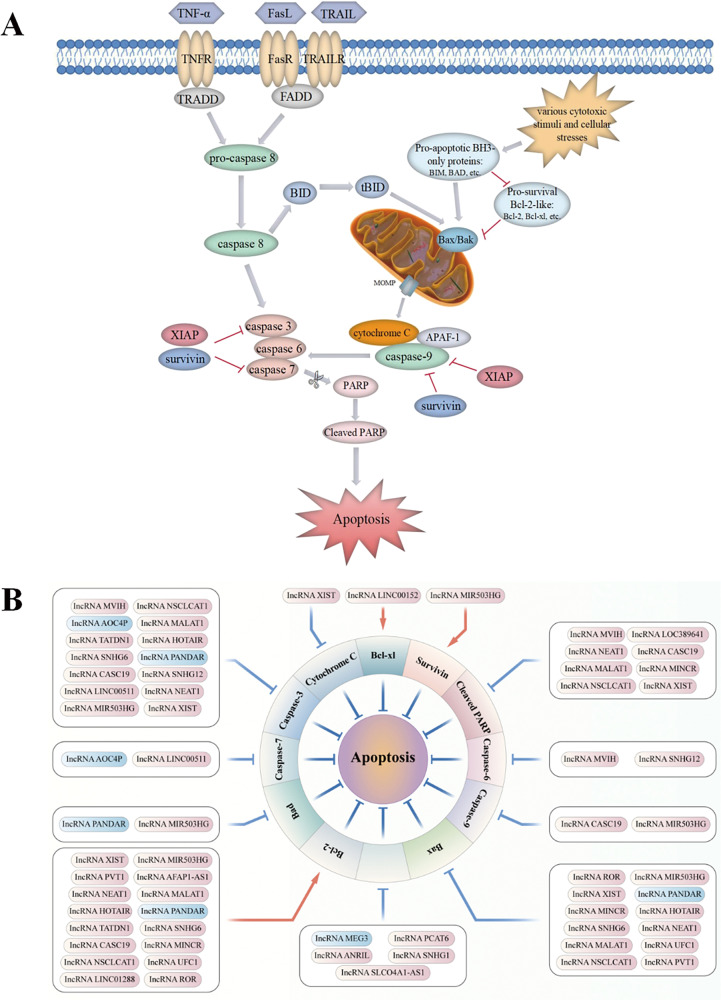
LncRNAs participate in the regulation of the apoptotic pathways in NSCLC. **A** Apoptosis can be initiated through two fundamental pathways: the death-receptor–mediated extrinsic pathway or the intracellular organelle-based intrinsic pathway. The extrinsic pathway can be activated by multiple extra-cellular death ligands, such as Fas ligand (FasL), TNF-α and TNF-related apoptosis-inducing ligand (TRAIL). The binding of ligands and their corresponding membrane death receptors then recruits death adaptor proteins, such as Fas-associated death domain (FADD) and TNF receptor-associated death domain (TRADD), to the death receptors. Subsequently, the oligomerized receptors and recruited adaptor proteins form the death-inducing signalling complex (DISC), which binds to pro-caspase-8 and triggers its activation. Active caspase-8 further proteolytically cleaves and promotes the activation of downstream apoptotic effector proteins caspase-3/6/7, and ultimately apoptosis. Various cytotoxic stimuli and cellular stresses activate the intrinsic pathway by inducing increased expression of BH3-only proteins (BIM, BAD, etc.) and inhibiting anti-apoptotic Bcl-2 proteins (Bcl-2, Bcl-xl, etc.). Activation of the pro-apoptotic effectors Bax and Bak ultimately causes mitochondrial outer membrane permeabilization (MOMP), which induces the release of cytochrome C. Cytochrome C engages apoptotic peptidase-activating factor 1 (APAF-1), subsequently triggers the activation of caspase-9 and in turn downstream effectors caspase-3/6/7, eventually contributing to apoptosis. In addition, active caspase-8 also engages the intrinsic apoptotic pathway indirectly through proteolytic activation of the pro-apoptotic protein BID into tBID. Moreover, the inhibitors of apoptosis proteins (IAPs), such as X-linked inhibitor of apoptosis protein (XIAP) and survivin, play a negative role in modulating both the extrinsic pathway and the intrinsic pathway by potently inhibiting key executioner caspase-3/7 activation and suppressing the initiator caspase-9 activity. **B** Various lncRNAs, including oncogenic lncRNAs (red box, high expression in NSCLC) and tumour-suppressive lncRNAs (blue box, low expression in NSCLC), participate in lung cancer-associated apoptosis by regulating apoptosis-related proteins.

Apoptosis is a non-inflammatory form of PCD initiated either via an extrinsic or intrinsic pathway, eventually inducing cellular demolition (Fig. 2A) [11]. A dynamic balance between pro-apoptotic and anti-apoptotic proteins is key to determining whether a cell maintains survival or undergoes apoptosis. However, dysregulation of apoptosis owing to imbalanced Bcl-2 family members, downregulation of caspases, overexpression of inhibitors of apoptosis proteins (IAPs) or impairment of death-receptor signalling is a common phenomenon exhibited in various cancers including NSCLC. These phenomena are responsible for not only tumorigenesis and cancer progression but also for resistance to multiple clinical therapies [12]. With regard to NSCLC pathogenesis, increased expression of the anti-apoptotic protein Bcl-2 and reduced levels of the pro-apoptotic protein Bax have been reported to be correlated with chemoresistance and tumour development [13, 14]. In addition, abnormalities in the activation of pivotal caspases, such as caspase 3/6/7/9, have also been observed in NSCLC progression. Moreover, studies have also suggested that caspase-8 is involved in the development of chemoresistance, which may serve as a potential target to improve chemosensitivity in NSCLC [15]. Poly (ADP-ribose) polymerase (PARP) plays a significant role in DNA damage repair and cell apoptosis, and dysregulated expression of PARP has been investigated as a possible biomarker and clinically useful target of lung cancer. In addition, several signalling pathways including STAT3, Wnt/β-catenin, NF-κB, PI3K/AKT, mTOR, MAPK/Slug, ROS, p53 and Nrf2 pathways, can regulate apoptosis through intrinsic or extrinsic mechanisms in the development of NSCLC [13, 16–20]. Explicit regulatory mechanisms of these pathways in apoptosis have been discussed in previous reviews. Accordingly, restoration of apoptosis by targeting both apoptotic pathways constitutes a feasible direction for developing promising anti-cancer drugs and enhancing the efficacy of clinical therapies.

### LncRNAs regulate apoptosis by influencing protein expression and signalling pathways in NSCLC

Numerous studies have demonstrated that lncRNAs participate in lung cancer-associated apoptosis by controlling various signalling regulatory proteins (Fig. 2B). Oncogenic lncRNAs are significantly upregulated in patients with NSCLC, which may advance lung carcinogenesis and cancer progression via inhibition of apoptosis by regulating the expression of associated proteins (Fig. 2B). Tang et al. [21] reported that overexpressed AFAP1-AS1 activated anti-apoptotic protein Bcl-2 expression and inhibited apoptosis in NSCLC while promoting cell proliferation. This effect was corroborated and reversed by silencing the expression of AFAP1-AS1, which downregulated the expression of Bcl-2. Another study demonstrated that higher expression of AFAP1‐AS1 was significantly correlated with larger tumour size, lymph node metastasis, higher tumour–node–metastasis (TNM) stage and worse overall survival in patients with NSCLC. Overexpression of AFAP1‐AS1 inhibited cell apoptosis but enhanced the proliferation and migration of lung cancer cells by partially repressing HBP1 expression through recruiting LSD1 to the HBP1 promoter regions [22]. The lncRNA MVIH is involved in drug resistance in NSCLC, and knockdown of MVIH restored drug sensitivity of cancer cells to cisplatin (DDP) by inducing apoptosis via upregulation of caspase-3, caspase-6 and cleaved PARP [23]. The lncRNA MINCR exerted inhibitory effects on apoptosis of NSCLC cells by activating the oncogene c-Myc and regulating the levels of its downstream apoptosis-associated proteins Bcl-2, Bax and cleaved-PARP-1, thus accelerating NSCLC progression [24]. Highly expressed lncRNA ANRIL was significantly related to TNM stages, tumour size and poor prognosis of patients in a study. Furthermore, ANRIL overexpression promoted NSCLC cell proliferation and repressed apoptosis by silencing KLF2 and p21 transcription through directly binding with EZH2 [25]. EZH2 inhibits the transcription of target genes by methylating histones, and its abnormal levels have been demonstrated to be correlated with the development of malignant tumours [26, 27]. In addition to ANRIL, other lncRNAs including HOTAIR, LINC00152, LINC00511, UFC1, PCAT6 and PVT1 bind to EZH2, thus epigenetically silencing gene expression involved in the regulation of NSCLC cell apoptosis. A study reported that EZH2 was highly expressed in NSCLC tissues and cells, and EZH2 overexpression inhibited the apoptosis of NSCLC cells via an increased expression of Bcl-2 and reduced levels of Bax and cleaved caspase-3. However, EZH2 downregulation caused by HOTAIR knockdown upregulated the expression of pro-apoptotic proteins, facilitating cell apoptosis in lung cancer [28]. Furthermore, LINC00152 inhibited the expression of tumour suppressor IL-24 by directly interacting with EZH2, which significantly increased the levels of Bcl-xl proteins, thus inhibiting apoptosis and promoting cell growth in lung adenocarcinoma [29]. Similarly, LINC00511 could serve as an oncogene repressing cell apoptosis and accelerating tumour growth partially by epigenetically suppressing the expression of p57 through binding with EZH2. Silencing of LINC00511 increased p57 expression and markedly augmented the caspase-3/7 activity, which facilitated NSCLC cell apoptosis and inhibited tumour proliferation [30]. The lncRNA UFC1 repressed cancer cell apoptosis and promoted NSCLC progression, partially exerting oncogenic effects through the recruitment of EZH2 to epigenetically inhibit PTEN expression and activate downstream AKt signalling. In UFC1-overexpressing H1299 cells, the level of Bcl-2 was significantly increased, whereas that of Bax was decreased [31]. LATS2, functioning as a tumour suppressor gene regulated by EZH2, was mutated in NSCLC, and overexpression of LATS2 induced cell apoptosis and inhibited NSCLC cell growth [32]. A study showed that the lncRNA PVT1 could repress cancer cell apoptosis and contribute to cell proliferation in lung adenocarcinoma partially through EZH2-mediated suppression of the LATS2/MDM2/P53 pathway [33]. Similar to PVT1, the oncogenic mechanism of the lncRNA PCAT6 was also reportedly involved in NSCLC progression [34].

However, tumour-suppressive lncRNAs, lowly expressed in NSCLC samples, play an inhibitory role in lung carcinogenesis and cancer progression by promoting cell apoptosis via modulation of the levels of associated proteins. For example, low expression of the lncRNA PANDAR predicted a poor prognosis of NSCLC and increased tumour cell proliferation by affecting the expression of the Bcl-2 protein family (Bax, Bad and Bcl-2) and caspase-3 via interaction with NF-YA at the transcriptional level [35].

In addition, multiple signalling pathways such as STAT3, p53, AKt/mTOR, Wnt/β-catenin and Hippo are also involved in the regulation of lncRNAs in apoptosis in NSCLC. Oncogenic lncRNAs can accelerate lung carcinogenesis and cancer progression by suppressing apoptosis through regulating related signalling pathways. The lncRNA LINC01288 was found to facilitate NSCLC progression both in vitro and in vivo by mediating the IL-6/STAT3 signalling pathway via stabilizing IL-6 mRNA. Activated STAT3 increased the level of downstream target Bcl-2, thus inhibiting NSCLC cell apoptosis [36]. Moreover, LOC389641 may activate STAT3 signalling by increasing the expression of EGFR and MET and therefore downregulate cleaved-PARP to inhibit cell apoptosis in lung adenocarcinoma [37]. In addition, the lncRNA MALAT1 promoted gefitinib resistance in NSCLC cells by inhibiting apoptosis through downregulating cleaved PARP and caspase-3 via STAT3 signalling activation [38]. Furthermore, the lncRNA XIST promoted cell proliferation and mediated chemoresistance of NSCLC to DDP by inhibiting apoptosis through interacting with SMAD2 and reducing p53 transcription, which subsequently modulated the expression of apoptosis-related proteins, including cytochrome c (cyto-c), Bax, Bcl-2 and caspase-3 (ref. [39]). The Akt/mTOR signalling pathway plays a significant role in regulating apoptosis, which is closely correlated with the drug resistance of NSCLC cells [40]. Studies have demonstrated that the lncRNA NEAT1 may contribute to paclitaxel resistance of NSCLC by suppressing apoptosis via activating Akt/mTOR signalling, with downregulation of cleaved PARP, cleaved caspase-3 and Bax proteins while upregulation of Bcl-2 (ref. [41]). Moreover, silencing of the lncRNA ROR was reported to improve the sensitivity of lung adenocarcinoma cells to DDP by inducing apoptosis through reducing Bcl-2 expression and increasing Bax levels via the PI3K/Akt/mTOR signalling pathway [42]. Furthermore, the lncRNA NSCLCAT1 inhibited cell apoptosis and accelerated NSCLC progression by inactivating the Hippo signalling pathway via inhibition of CDH1, with a decrease in the levels of Bax, cleaved caspase-3, and cleaved PARP and an increase in Bcl-2 expression [43].

Tumour-suppressive lncRNAs play an inhibitory role in lung carcinogenesis and cancer progression by enhancing cell apoptosis via modulation of related signalling pathways. The lncRNA MEG3 was significantly downregulated in NSCLC tissues, and its overexpression exerted anti-tumour effects, which may induce apoptosis and repress NSCLC development by upregulating the expression of tumour suppressor p53 and activating the p53 pathway [44]. In addition, upregulation of MEG3 could suppress tumour growth and cell proliferation in gemcitabine-resistant NSCLC by modulating PTEN signalling-mediated cell apoptosis [45]. Moreover, overexpression of the lncRNA AOC4P reduced c-Myc levels and increased caspase-3/7 activity, triggering apoptosis of NSCLC cells and inhibiting tumour proliferation by inactivating the Wnt/β-catenin pathway [46].

### LncRNAs regulate apoptosis as ceRNAs for sponging miRNAs in NSCLC

Substantial evidence suggests the involvement of miRNAs as critical gene regulators in the tumorigenesis of NSCLC. lncRNAs can function as sponges of miRNAs to regulate downstream gene expression, thereby modulating cell apoptosis and affecting cancer development. Wang et al. [47] confirmed elevated levels of lncRNA CASC19 and downregulation of miR-301b-3p in NSCLC cells and reported that CASC19 depletion accelerated cell apoptosis and suppressed cancer progression by repressing the expression of Bcl-2 and increasing the level of cleaved caspase-3, cleaved caspase-6 and cleaved PARP by sponging miR-301b-3p and modulating its downstream target LDLR. In addition, Wu et al. [48] demonstrated that the lncRNA PVT1 could competitively bind to miR181a-5p, contributing to an increased level of SP1, which was involved in the regulation of cell apoptosis and NSCLC cell growth. Moreover, it was reported that the lncRNA MIR503HG regulated the proliferation and apoptosis of NSCLC cells by directly interacting with miR-489-3p and miR-625-5p. MIR503HG knockdown remarkably upregulated Bax, Bad, cleaved caspase-3 and cleaved caspase-9 but downregulated Bcl-2 and survivin to promote apoptosis in cancer cells, thus suppressing NSCLC development [49]. Furthermore, the lncRNA SLCO4A1-AS1 repressed cancer cell apoptosis and drove NSCLC progression by increasing IKKα expression and activating the NF-κB signalling pathway by sequestering miR-223-3p [50]. The lncRNA SNHG1 contributed to NSCLC tumorigenesis and progression via the miR-101-3p/SOX9/Wnt/β-catenin axis, whereas depletion of SNHG1 induced cell apoptosis through inactivation of Wnt/β-catenin signalling, thereby inhibiting cancer proliferation [51]. Moreover, the lncRNA SNHG6 was demonstrated to inhibit apoptosis and promote proliferation in NSCLC by upregulating RSF1 through sponging miR-490-3p. However, SNHG6 knockdown repressed RSF1 to facilitate cell apoptosis by increasing the levels of Bax and cleaved caspase-3 and reducing the level of Bcl-2 (ref. [52]). The lncRNA XIST exhibited oncogenic properties in NSCLC, inhibiting cell apoptosis and promoting cancer malignancy partially mediated by miR-186-5p [53]. In addition, XIST could regulate the SOD2 signalling pathway by targeting miR-335 to modulate NSCLC cell apoptosis, and XIST downregulation enhanced the expression of caspase-3 and Bax and decreased the expression of Bcl-2 by reducing SOD2 levels [54]. Besides, XIST was reported to act as a miR-449a sponge to modulate the levels of Bcl-2 and cleaved PARP-1 by attenuating the endogenous function of miR-449a, which consequently influenced tumour progression of NSCLC [55]. The lncRNA MALAT1 was highly expressed in NSCLC cell exosomes and tissues, and its knockdown induced apoptosis by regulating Bcl-2/Bax protein expression and exerted anti-tumour effects by targeting EEF2 via miR-515-5p [56]. Moreover, MALAT1 could inhibit apoptosis and promote the malignant activity of NSCLC cells by regulating the miR-613/COMMD8 axis [57].

Growing evidence suggests a strong association between lncRNAs and NSCLC resistance to chemotherapy and radiation therapy. For example, the lncRNA TATDN1 could contribute to DDP resistance in NSCLC by inhibiting apoptosis via a decreased cleaved caspase-3 level and increased Bcl-2 expression through the miR-451/TRIM66 axis [58]. Furthermore, MALAT1 silencing repressed BRCA1 by binding to miR-146a and miR-216b, thus inducing apoptosis via upregulation of cleaved PARP1 and cleaved caspase-3 and sensitizing NSCLC cells to DDP [59]. Moreover, overexpression of AFAP1-AS1 has been observed in cisplatin (DDP)- or 5-fluorouracil (5-FU)-resistant NSCLC cells. Interfering with AFAP1-AS1 significantly inhibited cell proliferation and enhanced DDP- or 5-FU-induced apoptosis, alleviating chemotherapy resistance of cancer cells. This effect was mediated via suppression of RRM2/EGFR/AKT signalling through upregulation of miR-139-5p [60]. In addition, PVT1 is associated with radiotherapy resistance in NSCLC, and its knockdown promoted apoptosis owing to Bax upregulation and Bcl-2 downregulation and elevated the radiosensitivity of NSCLC cells by competitively binding to miR-424-5p to modulate CARM1 expression [61]. Moreover, studies have demonstrated that silencing of the lncRNA SNHG12 can not only enhance cancer cell apoptosis by increasing caspase-3 activity through upregulating miR-138 but also alleviate the resistance of NSCLC cells to anti-cancer agents (cisplatin, paclitaxel and gefitinib) by improving drug-induced apoptosis via upregulation of miR-181a, which suppresses the MAPK/Slug pathway and promotes caspase-3 and caspase-9 activities [62, 63].

## LncRNAs regulate autophagy in NSCLC

**Fig. 3:**
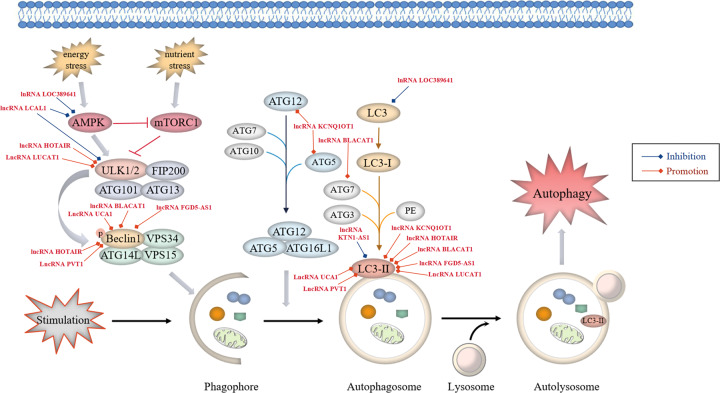
LncRNAs regulate the autophagy pathways in NSCLC. Autophagy starts with the formation of phagophores. The formation of unc-51-like kinase (ULK) complex from ULK1/2 kinase, ATG13, ATG101 and family-interacting protein FIP200, directly modulated by integrated signals from the mechanistic target of rapamycin complex 1 (mTORC1) and AMP-activated protein kinase (AMPK) signalling, triggers autophagic activity. Activated ULK1 can lead to the phosphorylation of Beclin1, thus activating class III PI3-kinase (VPS34) complex, which consists of VPS34, Beclin-1, VPS15 and ATG14L, primarily responsible for the nucleation of autophagosomal membrane. Two principal ubiquitination systems, ATG5-ATG12 and LC3 conjugation systems participate in autophagophore elongation and conversion into intact autophagosome. Respectively, ATG5 and ATG12 assemble into a complex, which then interacts with ATG16L1 to form a multimeric ATG12-ATG5-ATG16L1 conjugate that is on the outer surface of the autophagosomal membrane. Membrane-bound LC3-II is generated by the conjugation of microtubule-associated protein 1-light chain 3-I (LC3-I) to the lipid phosphatidylethanolamine (PE), providing a docking site for mounting autophagy cargo receptors that enable cargo loading into the autophagic vesicles. Various oncogenic lncRNAs (red font, high expression in NSCLC) participate in lung cancer-associated autophagy by regulating autophagy-related proteins.

Autophagy refers to a lysosome-mediated catabolic process dependent of multiple related proteins and signalling pathways, which plays a double-edged role in cancer (Fig. 3). On the one hand, autophagy restrains tumorigenesis by removing harmful cytotoxic agents to reduce stress injury, prevent genome damage and maintain cellular integrity [64]. In addition, defects in autophagy can promote inflammasome-mediated chronic inflammation and further induce the onset of lung cancer [65]. On the other hand, autophagy can be activated as a protective mechanism that helps to prevent intracellular metabolic and environmental stresses, such as nutrient starvation, energy shortage, hypoxia and cancer therapy, by regulating mitochondrial quality control and supply of nutrients required for cancer cell growth under nutrient-deprived conditions to facilitate cancer cell survival and tumour progression [66, 67]. Numerous studies have shown that protective autophagy is strongly correlated with resistance to anti-cancer drugs, and enhanced autophagy levels have been detected in patients with lung cancer with a poor prognosis [68]. Besides, it is reported that aberrant expression of autophagy-associated genes and proteins including unc-51-like kinase 1 (ULK1), ATG5, LC3B and p62 is involved in the onset and progression of NSCLC. In the lung tissues of NSCLC patients, LC3B expression was reduced, whereas the levels of p62, ULK1 and ATG5 were significantly increased, indicating that enhanced autophagy may be associated with the deterioration of lung cancer [69]. Furthermore, abnormal changes in the expression of genes such as epidermal growth factor receptor (EGFR), PTEN, LKB1 and AKT1 have been observed in NSCLC. These genes regulate EGFR-mediated signalling, which plays both promotive and suppressive roles in autophagic response via the RAS/RAF/MEK/ERK pathway to induce the serine phosphorylation of Beclin1 and the PI3K/AKT1 axis to inactive ULK1 by activating the negative modulator mTORC1, respectively [70, 71]. Furthermore, EGFR has emerged as a valuable target for anti-cancer therapy. Several studies have suggested that abnormal EGFR signalling participates in the induction of protective autophagy, which may be a promising approach to overcome resistance to anti-EGFR treatment in NSCLC [71]. In addition, p53 can regulate autophagic activity. Autophagy suppresses p53 by inhibiting AMPK activation and promotes tumorigenesis. Consequently, inhibition of p53 activates the transcription of autophagy-related genes, which further protects cells from apoptosis during hypoxia or nutrient starvation, resulting in tumour survival [13, 72]. Therefore, autophagy plays a pivotal role in the pathogenesis of NSCLC, and targeting autophagy is a promising approach to cancer treatment.

### LncRNAs regulate autophagy by influencing protein expression and signalling pathways in NSCLC

Multiple lncRNAs participate in lung cancer-associated autophagy by controlling various signalling regulatory proteins (Fig. 3). A study demonstrated that the lncRNA LOC389641 played an oncogenic role in lung adenocarcinoma through suppressing autophagy by reducing the expression of autophagy-related proteins, p-AMPK and LC3B via EGFR/MET/STAT3 signalling [37]. Oncogenic lncRNA HOTAIR has been reported to be correlated with drug resistance. In the study, Yang et al. [73] found that HOTAIR silencing reduced drug resistance of NSCLC cells to crizotinib by suppressing autophagy via inhibition of ULK1 phosphorylation, with decreased expression of Beclin-1 and LC3 II/I and increased p62 levels. ULK1 is a key regulator of autophagy, which plays a role in the regulation of drug resistance in NSCLC cells and cancer progression, suggesting that HOTAIR is a novel target for NSCLC therapy. Furthermore, it was reported that lung cancer-associated lncRNA 1 (LCAL1) was overexpressed in lung cancer tissues, which suppressed autophagic cell death, supporting cancer cell survival and proliferation by deactivating the AMPK/ULK1 signalling pathway [74].

### LncRNAs regulate autophagy as ceRNAs for sponging miRNAs in NSCLC

Huang et al. [75] demonstrated that the expression of lncRNA BLACAT1 was high in DDP-resistant NSCLC cells, which upregulated the autophagy-associated proteins ATG7 and Beclin1 and the LC3-II/LC3-I ratio to facilitate autophagy by sponging miR-17, thus resulting in the chemoresistance of NSCLC cells with an increased level of multidrug-resistance protein 1 (MRP1). Furthermore, elevated expression of the lncRNA FGD5-AS1 contributed to cancer cell progression by improving DDP resistance against NSCLC cells, exerting its tumour-promoting activity partially through induction of autophagy via the miR-140-5p/WEE1 axis. However, FGD5-AS1 depletion inhibited cell autophagy by blocking LC3-II/LC3-I and Beclin-1 expression while increasing p62 levels to hinder the progression of DDP-resistant cells in NSCLC [76]. Moreover, another study showed that the lncRNA LUCAT1 enhanced the resistance of NSCLC cells to DDP by regulating the miR-514a-3p/ULK1 axis. In addition to ULK1, overexpression of LUCAT1 further elevated the LC3-II/LC3-I ratio and decreased p62 levels to promote cancer cell autophagy [77]. The lncRNA PVT1 may function as a competing endogenous RNA for miR-216b and inhibit DDP sensitivity of NSCLC via induction of cell autophagy by increasing the LC3-II/LC3-I ratio and Beclin-1 protein abundance but reducing p62 levels [78]. As described by He et al., the lncRNA KCNQ1OT1 was responsible for resistance to stereotactic body radiotherapy (SBRT) in lung adenocarcinoma, directly promoting ATG5- and ATG12-dependent autophagic processes in cancer cells by sponging miR-372-3p, with a markedly elevated LC3-II/LC3-I ratio and decreased p62 levels [79]. In addition, it was reported that the lncRNA KTN1-AS1 promoted NSCLC progression by acting as a molecular sponge for miR-130a-5p and activating PDPK1, a negative modulator of autophagy, which downregulated autophagosomal LC3-II [80]. Aberrantly high expression of the lncRNA UCA1 has been reported in NSCLC, and UCA1 interference upregulated miR-185-5p to inhibit downstream β-catenin/TCF-4 activity, subsequently decreased Beclin-1 and LC3-II levels and enhanced p62 expression, thereby reducing autophagy in NSCLC cells and attenuating cancer proliferation [81].

## LncRNAs regulate pyroptosis in NSCLC

**Fig. 4:**
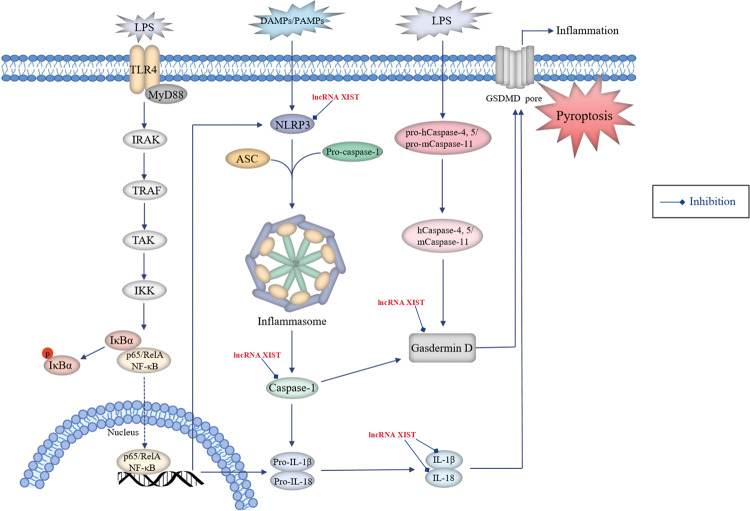
Roles of lncRNAs in the regulation of pyroptosis in NSCLC. Pyroptosis can be induced via the caspase-1-dependent canonical pathway and caspase-4,5 (for human)- or caspase-11 (for mouse)-mediated non-canonical pathway. In terms of the canonical pathway, damage-associated molecular patterns (DAMPs) or pathogen-associated molecular patterns (PAMPs) activate the inflammasome sensors NLRP3, which then recruit the effector pro-caspase-1 with the aid of the adaptor protein ASC to assemble inflammasomes, leading to the conversion of pro-caspase-1 into active caspase-1. Activation of NLRP3 inflammasome requires the involvement of the TLR4-mediated NF-κB pathway, which promotes the expression of NLRP3, pro-caspase-1, pro-IL-1β and pro-IL-18 proteins. Activated caspase-1 cleaves gasdermin D (GSDMD) and ultimately mediates pyroptosis. Besides, caspase-1 promotes the maturation of pro-inflammatory cytokines IL-1β and IL-18 by cleaving their precursor proteins, which trigger wide-ranging inflammatory responses. In the non-canonical pathway, human caspase-4 and -5 or their murine homologue caspase-11 can be directly activated by binding to lipopolysaccharide (LPS) and cleave GSDMD with efficiency similar to that of caspase-1, thereby inducing cell pyroptosis. Oncogenic lncRNAs (red font, high expression in NSCLC) participate in lung cancer-associated pyroptosis by regulating pyroptosis-related proteins.

Pyroptosis is a particular lytic and inflammatory form of PCD, which can be induced via the caspase-1-dependent canonical pathway and caspase-4,5 (for human)- or caspase-11 (for mouse)-mediated non-canonical pathway (Fig. 4) [82].

Evidence shows that the induction of pyroptosis via inflammasome/caspase-1 signalling stimulates a series of inflammatory cascades by releasing inflammatory mediators, which are strongly pertinent to tumorigenesis and cancer development [83]. Furthermore, pyroptosis has been reported to play a crucial role in suppressing the proliferation of tumour cells in vitro and tumour growth in vivo, suggesting that it is a promising strategy for NSCLC therapy [84]. The well-studied NLRP3 (NLR family pyrin domain-containing 3) inflammasome plays a dominant role in inducing pyroptosis and promoting inflammation, acting as an essential regulator of immune response that can be activated by chemotherapy or radiation treatment in various cancers, including NSCLC [85]. Therefore, the NLRP3 inflammasome is considered a promising therapeutic target for lung cancer prevention and treatment. For example, simvastatin suppressed NSCLC proliferation and migration by inducing cell pyroptosis through activating NLRP3 and caspase-1 and promoting the maturation of downstream IL-1β and IL-18 (ref. [86]). Several studies have indicated that mitochondrial reactive oxygen species (ROS) play a significant role in controlling tumour growth as crucial upstream regulators to trigger NLRP3 inflammasome activation and subsequent pyroptosis, wherein the signal transduction of ROS generation requires the involvement of NF-κB pathway, which increases the expression of NLRP3 and pro-caspase-1 proteins [87]. Moreover, p53 can inhibit cancer cell proliferation, tumour growth and NSCLC development as the key mediator activating pyroptosis via upregulation of NLRP3, ASC and cleaved caspase-1 (ref. [84]).

### LncRNAs regulate pyroptosis by influencing protein expression and signalling pathways in NSCLC

Several lncRNAs participate in lung cancer-associated pyroptosis by controlling various signalling regulatory proteins (Fig. 4). A study by Xu et al. reported that the lncRNA XIST had a positive impact on the resistance of NSCLC cells to DDP by directly interacting with SMAD2 and reducing its nuclear translocation, thus inhibiting cell pyroptosis via blockage of NLRP3 activation along with downregulated cleaved caspase-1, IL-1β, IL-18 and cleaved GSDMD [39].

### LncRNAs regulate pyroptosis as ceRNAs for sponging miRNAs in NSCLC

Liu et al. [54] reported that aberrant expression of lncRNA XIST could act as a sponge for miR-335, which participated in the regulation of NSCLC progression. XIST knockdown partially inhibited the development of NSCLC by targeting the miR-335/SOD2 signalling pathway and promoting downstream ROS generation, which induced pyroptotic cell death through activation of the NLRP3 inflammasome and increased levels of cleaved caspase-1, mature IL-1β and IL-18.

## LncRNAs regulate ferroptosis in NSCLC

**Fig. 5:**
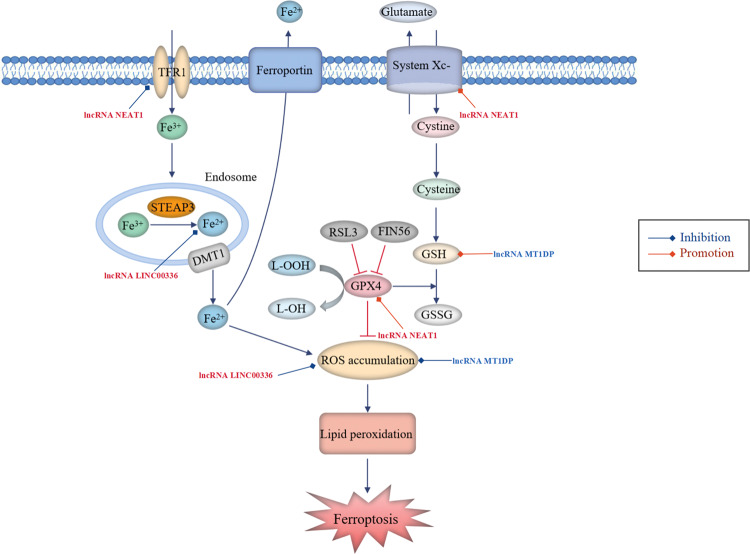
LncRNAs participate in the regulation of ferroptosis in NSCLC. Ferroptosis is initiated by blockade of the cellular antioxidant defences depending on glutathione (GSH). Cystine/glutamate antiporter system Xc- is responsible for transporting intracellular glutamate to the outside of the cell and transferring extra-cellular cystine into the cell. Subsequently, cystine is converted into cysteine for the biosynthesis of GSH. GPX4 converts GSH into oxidized glutathione (GSSG), concurrent with cytotoxic lipid peroxide (L-OOH) reduced to the corresponding alcohol (L-OH), thus reducing ROS accumulation. Cellular uptake of circulating iron (Fe^3+^) is mediated by TFR1 and intracellular iron can also be exported by ferroportin (FPN). The iron oxidoreductase six-transmembrane epithelial antigen of the prostate 3 (STEAP3) reduces Fe^3+^ to Fe^2+^, the latter is released from the endosome via divalent metal transporter 1 (DMT1) and then delivered into the unstable iron pool, thereby leading to ROS generation. Aberrant accumulation of ROS ultimately results in lipid peroxidation and ferroptosis. Oncogenic lncRNAs (red font, high expression in NSCLC) and tumour-suppressive lncRNAs (blue font, low expression in NSCLC) participate in lung cancer-associated ferroptosis by regulating ferroptosis-related proteins.

As a newly discovered non-apoptotic oxidative form of PCD, ferroptosis is primarily characterized by intracellular iron-dependent excessive accumulation of lipid ROS [88]. It is initiated by the blockade of the cellular antioxidant defence depending on glutathione (GSH), which is essential for restoring the intracellular redox homoeostasis upon ROS generation (Fig. 5) [89].

Glutathione peroxidase 4 (GPX4) plays a predominant role in the prevention of ferroptosis by decreasing cellular ROS levels and effectively repairing cell damage caused by lipid oxidation, and it was demonstrated to be high in NSCLC [90]. However, inactivation of GPX4 owing to GSH depletion and direct inhibition of GPX4 via RSL3- or FIN56-mediated GPX4 degradation lead to reduced antioxidant capacity and excessive production of lipid ROS, inducing ferroptosis as a result of unchecked lipid peroxidation [91]. Erastin, an exogenic small-molecule inducer of ferroptosis, not only promotes iron accumulation in cells by improving transferrin receptor 1 (TFR1) expression but also reduces cellular GSH levels by directly suppressing the activity of system Xc-, and it has been developed as an agent to treating various cancers, including NSCLC [92]. Furthermore, p53 has been reported to inhibit system Xc- uptake of cysteine through downregulating solute carrier family 7 member 11 (SLC7A11) by binding to nuclear factor erythroid 2-related factor 2 (NRF2), thereby promoting ferroptosis in cancer cell lines [93]. It has been demonstrated that repressing SLC7A11 transcription enhances the susceptibility of lung cancer cells to an oxidant insult, enabling cells to undergo ferroptosis-like cell death [94]. A study reported that NRF2 protected malignant cells from oxidative stress and chemotherapeutic agents, thus facilitating cancer progression [95]. Moreover, an elevated level of NRF2 rendered NSCLC cells insensitive to erastin-induced ferroptosis [96]. Therefore, suppression of NRF2 may result in an enhanced ROS-related oxidative stress level conducive to accelerating ferroptosis in NSCLC cells. Acyl-CoA synthetase long-chain family member 4 (ACSL4) is considered an essential pro-ferroptotic gene, and lower expression of ACSL4 has been associated with decreased ferroptosis in NSCLC cells [90, 97]. Several studies have shown oxidative stress resistance in NSCLC and weak sensitivity of tumour cells to ferroptosis, suggesting that ferroptosis has an important influence on the occurrence and progression of NSCLC [98]. Moreover, it was demonstrated that ferroptosis participated in modulating the sensitivity of NSCLC cells to chemotherapy and radiotherapy [99, 100]. Therefore, targeting ferroptosis is a novel approach to developing therapeutic strategies for NSCLC.

### LncRNAs regulate ferroptosis by influencing protein expression and signalling pathways in NSCLC

Several lncRNAs participate in lung cancer-associated ferroptosis by controlling various signalling regulatory proteins (Fig. 5). Wu et al. [90] found that ACSL4 was positively correlated with ferroptosis induced by erastin in NSCLC cells and demonstrated that the lncRNA NEAT1 played a negative regulatory role in ferroptosis and could repress the ferroptosis sensitivity of NSCLC cells to erastin by directly binding to ACSL4 and reducing its protein expression. In addition, NEAT1 overexpression increased the levels of SLC7A11 and GPX4 but downregulated TFR1, which was consistent with the mechanism of ferroptosis inhibition. Therefore, targeting NEAT1 may be a potential therapeutic strategy for NSCLC.

### LncRNAs regulate ferroptosis as ceRNAs for sponging miRNAs in NSCLC

Wang et al. [101] identified that the nuclear lncRNA LINC00336 was upregulated in lung cancer, which accelerated cancer cell growth and proliferation by suppressing ferroptosis. Mechanistically, overexpression of LINC00336 reduced intracellular lipid ROS accumulation, iron and Fe^2+^ concentrations by functioning as an endogenous sponge of miR-6852 in a cystathionine-β-synthase (CBS)-dependent manner. In addition, Gai et al. [96] reported that overexpression of the tumour suppressor lncRNA MT1DP sensitized erastin-induced ferroptosis with reduced cellular GSH levels and elevated lipid ROS generation through downregulating NRF2 by stabilizing miR-365a-3p in NSCLC cells.

## Potential chemical drugs and herbal medicine targeting lncRNAs in PCD for the treatment of NSCLC

Targeting lncRNAs displays great potential in NSCLC therapy. Recent studies have shown that some drugs can act on lncRNAs to regulate PCD in cancer cells, thus suppressing NSCLC development. To date, chemotherapeutic drugs are the most commonly used therapeutic agents for NSCLC. Paclitaxel (PTX) serves as a first-line chemotherapeutic drug for advanced NSCLC in clinical settings. A study demonstrated that PTX treatment (10 μM) inhibited A549 cell proliferation and partially induced cell apoptosis by upregulating lncRNA MEG3 and activating the p53 pathway [102]. In addition, another study indicated that nedaplatin (2 mg/mL), a functional analogue of the anti-cancer drug cisplatin, could restore the chemotherapeutic sensitivity of cisplatin-resistant A549 and H1650 cells. The underlying mechanism may involve the induction of apoptosis by regulating targets that included caspase-3, caspase-6 and cleaved PARP through downregulation of lncRNA MVIH, thus providing a rationale for nedaplatin use in the treatment of NSCLC [23].

Conventional herbal medicine also exhibits good anti-cancer effects, which is widely used as an auxiliary or alternative medication for cancer therapy in China [103]. Polyphyllin I (0, 0.4, 0.8 and 1.2 μmol/L), a steroidal saponin extracted from the rhizome of the Chinese herbal medicine *Paris polyphylla* with strong inhibitory effects on various cancers, was reported to induce apoptosis by downregulating Bcl-2 and upregulating Bax and cleaved caspase-3 in a dose-dependent manner through impeding the STAT3/HOTAIR/EZH2 signalling axis, thus inhibiting A549 and NCI-H358 cell growth [28]. Moreover, Yang et al. [38] suggested that treatment with polyphyllin I (0.1, 1, 2, 4, 6, 8 and 10 μg/ml in vitro; 5 mg/kg/d for 4 weeks in vivo) suppressed the viability of gefitinib-resistant PC-9-ZD cells in vitro and tumour growth in PC-9-ZD xenograft in vivo in a dose-dependent manner. This anti-tumour activity was mediated through induction of apoptosis via an increase in the level of cleaved PARP and caspase-3 by downregulating lncRNA MALAT1 and inactivating STAT3 signalling. Curcumin, a flavonoid compound widely found in various medicinal herbs, has been reported to inhibit multiple human cancer types, especially lung cancer. It was demonstrated that curcumin (50 and 100 μM in vitro; 100 mg/kg/d for 3 weeks in vivo) significantly suppressed gemcitabine-resistant NSCLC cell proliferation in a concentration-dependent manner and restrained tumour growth by promoting cell apoptosis through upregulation of lncRNA MEG3-mediated PTEN signalling [45]. Xiaoji decoction (XJD) is a Chinese medicinal formulation that has been used for the treatment of lung cancer for decades. It is comprised of *Psoralea corylifolia* Linn, *Coriolus versicolor*, *Astragalus membranaceus* (Fisch) Bunge, *Curcumae Rhizomae*, *Hedyotis diffusa* Willd, *Buthus martensii* Karsch, *Scolopendra subspinipes* and *Rheum palmatum* L. In a study by Wu et al., XJD (40 mg/mL in vitro; 13.4 g/kg/d for 25 days in vivo) enhanced the inhibitory effects of DDP not only on A549 and H1975 cell growth but also on lung tumour size by inducing a high magnitude of cell apoptosis via downregulation of SP1 through regulating the reciprocal interaction between lncRNA PVT1 and miR181a-5p [48].

## Conclusion and future prospects

Recent studies have recognized that aberrant expression of lncRNAs is strongly correlated with the initiation and development of multiple cancers, including NSCLC. Abnormal regulation of PCD, including apoptosis, autophagy, pyroptosis and ferroptosis, has been demonstrated to play a critical role in the pathogenesis of NSCLC. LncRNAs can regulate PCD and relevant pathways in lung cancer cells and exert oncogenic or anti-carcinogenic effects by either directly influencing protein expression or functioning as a sponge of miRNAs (Table 1). As previously described, a considerable number of lncRNAs have been implicated in lung carcinogenesis and have been associated with resistance to anti-cancer drugs, thereby representing a potential value as predictive biomarkers for chemotherapeutic sensitivity and clinical prognosis of cancer. For example, circulating lncRNA SOX2OT and ANRIL, which were markedly overexpressed in the serum of patients with NSCLC, have been evaluated as ideal biomarkers for diagnosing NSCLC and predicting the overall survival rate of NSCLC patients, yielding superior sensitivity and specificity with remarkable potential in distinguishing patients with cancer from controls [104]. Therefore, an in-depth understanding of the relationship between lncRNAs and PCD may provide novel insights into the pathogenic molecular mechanisms involved in NSCLC, and targeting lncRNAs is expected to be a promising strategy for lung cancer treatment.

**Table 1:** Effects of lncRNAs on PCD in NSCLC.

Programmed cell death type	LncRNA	Expression in NSCLC	Function	Pathway	Target	Ref.
Apoptosis	AFAP1-AS1	↑	Oncogene		Bcl2 ↑	21
					LSD1 ↑ HBP1 ↓	22
				RRM2/EGFR/AKT pathway	RRM2, EGFR, AKT ↑ miR-139-5p ↓	60
	MVIH	↑	Oncogene		caspase-3, caspase-6, cleaved PARP ↓	23
	MINCR	↑	Oncogene		c-Myc, Bcl-2 ↑ Bax, cleaved PARP1 ↓	24
	PANDAR	↓	Tumour suppressor		NF-YA, Bcl2 ↑ Bax, Bad, cleaved-caspase-3 ↓	35
	ANRIL	↑	Oncogene		EZH2 ↑ KLF2, p21 ↓	25
	HOTAIR	↑	Oncogene		EZH2, Bcl-2 ↑ Bax, cleaved-caspase-3 ↓	28
	LINC00152	↑	Oncogene		EZH2, Bcl-xl ↑ IL24 ↓	29
	LINC00511	↑	Oncogene		EZH2 ↑ p57, caspase-3/7 ↓	30
	UFC1	↑	Oncogene	Akt pathway	EZH2, p-Akt, Bcl-2 ↑ PTEN, Bax ↓	31
	PVT1	↑	Oncogene	MDM2/p53 pathway	EZH2, MDM2 ↑ LATS2, P53 ↓	33
					SP1 ↑ miR181a-5p ↓	48
					CARM1, Bcl-2 ↑ miR-424-5p, Bax ↓	61
	PCAT6	↑	Oncogene		EZH2 ↑ LATS2 ↓	34
	LINC01288	↑	Oncogene	IL-6/STAT3 pathway	IL-6, p-STAT3, Bcl2 ↑	36
	LOC389641	↑	Oncogene	EGFR/MET/STAT3 pathway	EGFR, MET, p-STAT3 ↑ cleaved-PARP ↓	37
	MALAT1	↑	Oncogene	STAT3 pathway	p-STAT3 ↑ cleaved-PARP, cleaved-caspase-3 ↓	38
					EEF2, Bcl2 ↑ miR-515-5p, Bax ↓	56
					COMMD8, Bcl-2 ↑ miR-613, Bax ↓	57
					BRCA1 ↑ miR-216b, miR-146a, cleaved PARP1, cleaved-caspase-3 ↓	59
	MEG3	↓	Tumour suppressor	p53 pathway	p53 ↓	44
				PTEN pathway	PTEN ↓	45
	XIST	↑	Oncogene	SMAD2/p53 pathway	Bcl-2 ↑ SMAD2, p53, Cytochrome C, Bax, Caspase-3 ↓	39
					Bcl-2 ↑ miR-186-5p ↓	53
				SOD2/ROS pathway	SOD2, Bcl-2 ↑ miR-335, ROS, Bax, Caspase-3 ↓	54
					Bcl-2 ↑ miR-449a, cleaved-PARP-1 ↓	55
	NEAT1	↑	Oncogene	Akt/mTOR pathway	p-Akt, p-mTOR, Bcl-2 ↑ Bax, cleaved-PARP, cleaved-caspase-3 ↓	41
	ROR	↑	Oncogene	PI3K/Akt/mTOR pathway	p-PI3K, p-Akt, p-mTOR, Bcl-2 ↑ Bax ↓	42
	AOC4P	↓	Tumour suppressor	Wnt/β-catenin pathway	β-catenin, c-Myc ↑ caspase 3/7 ↓	46
	NSCLCAT1	↑	Oncogene	The Hippo pathway	TAZ, p-TAZ, YAP1, p-YAP1, Bcl-2 ↑ CDH1, Lats, Mst1, Bax, cleaved caspase-3, cleaved PARP ↓	43
	CASC19	↑	Oncogene		LDLR, Bcl2 ↑ miR-301b-3p, cleaved-caspase-3, cleaved-caspase-6, cleaved-PARP ↓	47
	MIR503HG	↑	Oncogene		Bcl-2, Survivin ↑ miR-489-3p, miR-625-5p, Bax, Bad, cleaved-caspase-3, cleaved-caspase-9 ↓	49
	TATDN1	↑	Oncogene		TRIM66, Bcl-2 ↑ miR-451, cleaved-caspase-3 ↓	58
	SLCO4A1-AS1	↑	Oncogene	NF-κB pathway	IKKα ↑ miR-223-3p ↓	50
	SNHG1	↑	Oncogene	Wnt/β-catenin pathway	SOX9, Wnt/β-catenin ↑ miR-101-3p ↓	51
	SNHG12	↑	Oncogene		miR-138, caspase-3 ↓	62
				MAPK/Slug pathway	MAPK1, p-MAPK1, MAP2K1, p-MAP2K1, Slug ↑ miR-181a, caspase-3, caspase-9 ↓	63
	SNHG6	↑	Oncogene		RSF1, Bcl-2 ↑ miR-490-3p, Bax, cleaved-caspase-3 ↓	52
Autophagy	LOC389641	↑	Oncogene	EGFR/MET/STAT3 pathway	EGFR, MET, p-STAT3 ↑ p-AMPK, LC3B ↓	37
	HOTAIR	↑	Oncogene	ULK1 pathway	p-ULK1, Beclin-1, the ratio of LC3-II/I ↑ p62 ↓	73
	LCAL1	↑	Oncogene	AMPK/ULK1 pathway	p-AMPKα, p-ULK1 ↓	74
	BLACAT1	↑	Oncogene	ATG7 pathway	ATG7, LC3-II/LC3-I, MRP1, Beclin1 ↑ miR-17 ↓	75
	FGD5-AS1	↑	Oncogene		WEE1, Beclin-1 LC3-II/LC3-I ↑ miR-140-5p, p62 ↓	76
	LUCAT1	↑	Oncogene	ULK1 pathway	ULK1, LC3-II/LC3-I ↑ miR-514a-3p, soluble p62 ↓	77
	PVT1	↑	Oncogene		Beclin-1, LC3-II/LC3-I ↑ miR-216b, p62 ↓	78
	KCNQ1OT1	↑	Oncogene		ATG5, ATG12, LC3-II/LC3-I ↑ miR-372-3p, p62 ↓	79
	KTN1-AS1	↑	Oncogene	PDPK1 pathway	PDPK1 ↑ miR-130a-5p, LC3-II ↓	80
	UCA1	↑	Oncogene	β-catenin pathway	β-catenin, TCF-4, Beclin-1, LC3-II ↑ miR-185-5p, p62 ↓	81
Pyroptosis	XIST	↑	Oncogene	SMAD2/NLRP3 pathway	SMAD2, NLRP3, cleaved caspase-1, IL-1β, IL-18, cleaved GSDMD ↓	39
				SOD2/ROS/NLRP3 pathway	SOD2 ↑ miR-335, ROS, NLRP3, cleaved caspase-1, IL-1β, IL-18 ↓	54
Ferroptosis	NEAT1	↑	Oncogene		SLC7A11, GPX4 ↑ ACSL4, TFR1 ↓	90
	LINC00336	↑	Oncogene		CBS ↑ miR-6852, iron, ROS ↓	101
	MT1DP	↓	Tumour suppressor	NRF2 pathway	NRF2, GSH ↑ miR-365a-3p, ROS ↓	96

Multiple RNA interference technologies, such as small interfering RNAs (siRNA), short hairpin RNA (shRNA) and antisense oligonucleotides (ASOs), exhibit the most intriguing approach to selectively depleting or knocking down the target oncogenic lncRNAs. For instance, MALAT1 knockdown through siRNA has been reported to block cancer cell proliferation and reduce tumour growth in NSCLC by downregulating COMMD8 [57]. He et al. [105] demonstrated that silencing CCAT2 with shRNA inhibited the proliferation, invasion and tumour formation of NSCLC cisplatin-resistant cells. In addition, Gong et al. [106] constructed MALAT1-specific ASO and nucleus-targeting TAT peptide co-functionalized Au nanoparticles, namely, ASO-Au-TAT NPs, to precisely target and degrade nuclear MALAT1, thus effectively preventing lung cancer metastasis. It is noteworthy that compared with free ASO, ASO conjugated with Au-TAT NPs represented much higher nucleus-specific delivery efficiency and more efficiently inhibited target lncRNA. Owing to low stability and poor penetration into the nucleus, free ASO exhibited limited anti-metastasis capacity in vivo even at a very high dose [107]. Therefore, this novel nanostructure has great potential for being used in cancer metastasis therapy in the future. The expression of tumour-suppressive lncRNAs was reported to be lower in lung cancer specimens than in healthy tissues. Accordingly, restoring the normal expression levels of these lncRNAs may provide a promising therapeutic approach beneficial to patients with NSCLC. For example, Li et al. [46] demonstrated that AOCP4 was downregulated in NSCLC samples, and upregulation of AOCP4 via transfection of pcDNA suppressed cell viability and invasion in NSCLC but induced cell apoptosis by inactivating the Wnt/β-catenin pathway. However, there are several issues concerning lncRNA-based therapies in clinical practice. It is important to develop a reliable delivery system to safeguard the stability of lncRNA vectors in the bloodstream and improve the efficiency of selectively attacking the tumour targets. In addition, deciding an appropriate dosage is necessary to ensure the safety and effectiveness of therapeutic lncRNAs, which requires further investigation [6, 108].

To date, a few chemical drugs and herbal medicines have been reported to target lncRNAs in PCD for NSCLC treatment, whose regulatory mechanisms mostly focus on cell apoptosis. Therefore, developing related anti-cancer medication, especially herbal medicines, which have an excellent prospect, will help to establish a more comprehensive clinical approach to improving the therapeutic outcomes of NSCLC, with the final goal to benefit the patients.

## Data Availability

Data sharing is not applicable to this article as no datasets were generated or analysed during the current study.
